# Systems-level analysis of local field potentials reveals differential effects of lysergic acid diethylamide and ketamine on neuronal activity and functional connectivity

**DOI:** 10.3389/fnins.2023.1175575

**Published:** 2023-05-23

**Authors:** Azat Nasretdinov, Sebastian A. Barrientos, Ivani Brys, Pär Halje, Per Petersson

**Affiliations:** ^1^The Group for Integrative Neurophysiology, Department of Integrative Medical Biology, Umeå University, Umeå, Sweden; ^2^The Group for Integrative Neurophysiology and Neurotechnology, Department of Experimental Medical Science, Lund University, Lund, Sweden; ^3^Postgraduate Program in Psychology, Health, and Biological Sciences, Federal University of Vale do São Francisco (UNIVASF), Petrolina, Brazil

**Keywords:** psychedelics, dissociative anesthetics, *in vivo*, neurophysiology, LFP

## Abstract

Psychedelic substances have in recent years attracted considerable interest as potential treatments for several psychiatric conditions, including depression, anxiety, and addiction. Imaging studies in humans point to a number of possible mechanisms underlying the acute effects of psychedelics, including changes in neuronal firing rates and excitability as well as alterations in functional connectivity between various brain nodes. In addition, animal studies using invasive recordings, have suggested synchronous high-frequency oscillations involving several brain regions as another key feature of the psychedelic brain state. To better understand how the imaging data might be related to high-resolution electrophysiological measurements, we have here analyzed the aperiodic part of the local field potential (LFP) in rodents treated with a classic psychedelic (LSD) or a dissociative anesthetic (ketamine). In addition, functional connectivity, as quantified by mutual information measures in the LFP time series, has been assessed with in and between different structures. Our data suggest that the altered brain states of LSD and ketamine are caused by different underlying mechanisms, where LFP power shifts indicate increased neuronal activity but reduced connectivity following ketamine, while LSD also leads to reduced connectivity but without an accompanying change in LFP broadband power.

## Introduction

In recent years, several psychedelic substances, such as DMT, 5-MeO-DMT, LSD and psilocybin, but also dissociative anesthetics with hallucinogenic properties, like ketamine, have been suggested to have therapeutic potential for the treatment of mood and anxiety disorders, neurodegenerative disorders, alcohol-use disorder, and various substance-use disorders, even in patients who are otherwise treatment-resistant (see e.g., Mithoefer et al., 2016; Johnson and Griffiths, 2017; Davis et al., 2019; Rosenblat et al., 2019; Bahji et al., 2020; de Gregorio et al., 2021; Lowe et al., 2021).

As a result, several trials have been initiated to assess tolerability and efficacy of these compounds for clinical use (>100 clinical trials are currently registered^1^ for psilocybin or ketamine alone). However, there is at present little understanding of the mechanistic actions of these compounds making this an urgent knowledge gap to bridge given the rapid development toward wider clinical use.

Using functional neuroimaging of glucose metabolism (PET) or changes in blood flow (fMRI), different changes in brain activity following drug administration has been characterized, pointing to possible mechanism involving, for example, deficiencies in thalamic gating of sensory input (Preller et al., 2018, 2019), or decreased functional connectivity between nodes of the default mode network (Palhano-Fontes et al., 2015; Doss et al., 2022). Other human studies have used electroencephalogram (EEG) and magnetoencephalography (MEG) to assess brain activity with higher temporal resolution, revealing reduced synchronized oscillatory activity, which may point to a decreased functional connectivity between certain brain structures (Muthukumaraswamy et al., 2013; Carhart-Harris et al., 2016). For high-resolution recording of physiological processes in neuronal circuits, animal experiments using invasive recording techniques are, however, a necessary complement. The number of published *in vivo* studies characterizing neurophysiological effects of hallucinogenic drugs are still relatively few, but a handful of pioneering papers have provided important pieces of information. Recording the firing rate of individual neurons, it has for example, been demonstrated that serotonin 2A agonists tend to decrease spiking activity in the orbitofrontal cortex, anterior cingulate cortex and the motor cortex of rodents (Wood et al., 2012; Rangel-Barajas et al., 2017), whereas NMDA antagonists instead tend to excite cells in prefrontal cortex (Suzuki et al., 2002; Jackson et al., 2004; Homayoun and Moghaddam, 2007; Wood et al., 2012; Furth et al., 2017; Amat-Foraster et al., 2019). Notably, however, populations of inhibitory interneurons may be differently affected compared to the excitatory network of principal cells (Suzuki et al., 2002; Jackson et al., 2004; Homayoun and Moghaddam, 2007; Wood et al., 2012; Furth et al., 2017; Amat-Foraster et al., 2019). Moreover, analyses of synchronized network activity have indicated a tendency for cognitive-limbic brain structures to engage in high-frequency oscillations (HFOs) at frequencies well above 100 Hz after NMDA antagonist treatment (Hunt et al., 2006, 2011; Hakami et al., 2009; Nicolás et al., 2011; Kulikova et al., 2012), as well as after serotonin 2A agonists treatment (Olszewski et al., 2013; Hansen et al., 2019). However, obtaining detailed neurophysiological measurements of changes in brain activity on a detailed scale, but still probing the widely distributed circuits thought to be involved in higher cognitive functions has proven a daunting task. For this reason, bridging the scales between cellular recordings to systems-level imaging data remains an unsolved challenge, severely limiting our understanding of the mechanistic action of these compounds and their possible use in novel therapies.

As a first step toward systems-level mapping of high-resolution neurophysiological data, we have here performed large-scale multi-structure microelectrode recordings in twelve rats treated with LSD, ketamine and amphetamine. Analyses were here focused on neuronal population features in the neurophysiological signals that are thought to correlate to neuronal activity at the circuit level (and presumably also the BOLD signal analyzed in fMRI experiments). In particular, we chose to investigate the aperiodic component of the local field potentials (LFPs). The rationale being that while the LFP signal is thought to be dominated by synchronized dendritic currents (Herreras, 2016), the spiking of individual excitatory and inhibitory neurons likely give a relatively larger contribution to the population LFP under desynchronized, non-oscillatory conditions and has, as such, been linked to the fMRI signal [Manning et al., 2009; Kahn et al., 2013; for a complementary in depth analysis of the oscillatory components we would like to refer the reader to Brys et al. (2022)]. In specific, we have here, first, analyzed the frequency-dependent broad-band power changes in voltage fluctuations measured between pairs of microelectrodes and, second, assessed functional connectivity between thousands of electrode pairs located in same or different brain structures, using the Gaussian copula mutual information (MI) metrics in time series analyses (Ince et al., 2017).

## Materials and methods

### Animals

Eleven female and one male Sprague-Dawley rats of 250–350 g were used (Taconic, Denmark) in this study. All procedures were approved in advance by the Malmö/Lund ethical committee of animal experiments.

### Construction and implantation of electrode arrays

Microelectrode arrays with 128 individual tungsten wires (33 μm in diameter and insulated with formvar; California Fine Wire, Grover Beach, CA, United States) were built and implanted as previously described (Ivica et al., 2014; see also Supplementary Figure 1). Three screws anchored in the occipital bone were connected to a silver wire and used as a ground for the recording system. Animals were allowed to recover for at least 1 week after implantation.

### Pharmacological treatments

Animals were placed in a circular open field arena, 75 cm in diameter, and 50 cm high acrylic walls. After about 60 min of baseline recording, the animals were intraperitoneally injected with a solution of either LSD (lysergic acid diethylamid, 0.3 mg/kg, Lipomed AG, Arlesheim, Switzerland), ketamine [Ketaminol, 25 or 50 mg/kg (the dose was adjusted individually for each rat to a level just below the point where the animal became ataxic), Intervet AB, Stockholm, Sweden] or amphetamine (d-amphetamine hydrochloride, 4 mg/kg, Tocris, Bristol, United Kingdom) and recorded for another 60–120 min. Data were averaged over -35 to -5 min in relation to the time of drug injection for baseline measurements and 30–60 min for on-drug measurements. All animals were tested on the three above-mentioned drugs in a pseudo-randomized order, with at least 48 h of rest between consecutive experiments.

### Signal acquisition

Local field potentials were recorded with the Neuralynx multichannel recording system using a unity gain preamplifier (Neuralynx, Bozeman, MT, United States) or with the OpenEphys acquisition system (Siegle et al., 2017) using 4 Intan RHD2132 amplifier boards with on-board AD-converters and accelerometers (Intan technologies, CA, USA). Signals were processed and analyzed offline using Matlab Release 2020b (Mathworks, Natick, MA, United States).

### Determining the anatomical location of recording electrodes

Electrode positions were verified post-mortem in seven out of the twelve animals using micro computed tomography (CT) based image stacks (Censoni et al., 2022; for the remaining five rats where CT data were lacking, target coordinates were instead used). A total of 50 unique anatomical labels were attributed to the electrode tips in this way and they were further grouped into nine broader regions based on functional similarity.

### LFP power spectral densities

To emphasize local sources of the measured electrical potential, bipolar LFP time series were computed from all individual pairs of electrodes from the same structure, after which the averaged spectrograms were calculated for each structure. Median power spectra were then calculated at time intervals of -32 to -2 min and 30 to 60 min relative to the time of drug treatment. Power spectra for aperiodic activity were separated from periodic activity using the Fitting Oscillations and One-Over-F algorithm (Donoghue et al., 2020). The corresponding aperiodic spectra for baseline and post-treatment episodes were approximated by a function of the form *y* = 10*^A^*/f*^B^*, where parameters A and B reflect the offset (uniform shift of power across frequencies) and the slope (aperiodic exponent reflecting the pattern of aperiodic power across frequencies), respectively.

### Connectivity analysis

Gaussian copula MI was used to calculate the measure of connectivity between all pairs of electrodes (Ince et al., 2017). MI was calculated for non-filtered LFP time-series (raw monopolar data down-sampled to 1 or 2 kHz) in 2 s windows at -20 and -5 min before and at 45 and 60 min after drug treatment. The median of 450 values at baseline and after treatment was then calculated, followed by the calculation of relative change after treatment/baseline for each electrode pair. The values obtained were checked against the corresponding ratio changes between two 15 min baseline periods.

### Statistical analysis

Statistical analysis was performed using the non-parametric Wilcoxon signed rank or rank sum test. The normality assumption was assessed using the Kolmogorov–Smirnov test. Since the test rejected the corresponding null hypothesis, non-parametric statistics were used. The results of the comparison with *p*-value >0.05 were considered not significant.

## Results

### Spatial mapping of microelectrode recordings

**FIGURE 1:**
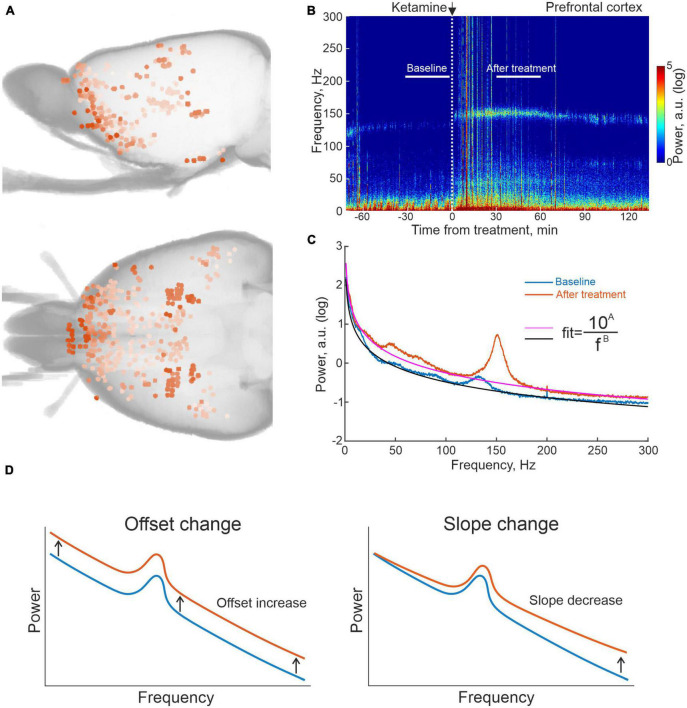
Summary of reconstructed recording locations and one example of local field potential (LFP) data from prefrontal cortex (PFC) on ketamine. **(A)** 3D reconstruction of recording sites from computed tomography (CT) scans of seven of the recorded rats. **(B)** Example of an averaged spectrogram representing the differential LFP signal from pairs of electrodes located in PFC in conjunction with ketamine treatment, and **(C)** the corresponding time-averaged spectra for the 30 min time periods indicated in 1B. White vertical dashed line in **(B)** marks time of ketamine injection; black and magenta lines for the two spectra in **(C)** represent fits of the form (*y* = 10^A^/f^B^) to the non-oscillatory part of the data (i.e., disregarding the oscillatory activity represented by the humps, e.g., HFOs at 130–160 Hz). **(D)** Schematic representation of spectral changes in offset and slope corresponding to increases in the fitted parameters (A,B), respectively.

Toward the aim of characterizing the brain-wide physiological responses to the investigated compounds with the spatial resolution offered by microelectrode recording techniques, we here designed large-scale multi-structure recording arrays that targeted both cognitive-limbic and sensorimotor related structures in the cortico-basal ganglia-thalamic circuits and the medial septal area. In total 933 sites were recorded in the twelve animals (see Figure 1A for maps of the spatial distribution of electrode tips from seven of the recorded rats, for which full CT reconstructions were made). Following functional grouping of recording locations and exclusion of regions with insufficient number of recordings across animals (at least six recordings from least three animals in the same structure), the following broader cognitive-limbic and frontal regions were included in the further analyses: olfactory cortex and associated structures (OlfC), orbitofrontal cortex (OFC), prefrontal cortex (PFC), ventral striatum (vStr), the septal area (SepA), and integrative nuclei of the thalamus (IntTh), as well as a number of sensorimotor regions: primary sensorimotor cortex (SenC), the dorsal striatum (dStr), the temporal association area (TemAA), and sensory nuclei of the thalamus (SenTh). Recordings were carried out in an open-field arena where the animals were free to spontaneously move around during the experiments. After a period of adaptation to the recording environment, a baseline was obtained for a period of 30 min, during which the animals were typically rather immobile. After this, each animal was injected with one of the following three compounds (LSD 0.3 mg/kg, ketamine 25 or 50 mg/kg or amphetamine 4 mg/kg; the substances were administered in different experiments with at least 2 days between subsequent recording sessions in a pseudo randomized order), after which another 30 min period was recorded in the drug-treated state (30–60 min after injection for all three drugs). LFPs were recorded from all 933 electrodes, and to ensure measurements represented local voltage fluctuations, the LFP signals from pairs of neighboring electrodes were subtracted, eliminating any potential contributions from external sources (Halje et al., 2012; Herreras, 2016). In Figure 1B, an example differential recording from a pair of electrodes located in PFC, in conjunction with systemic ketamine treatment, is shown illustrating the experimental design and typical changes arising in LFP spectral content [in (B), spectrogram for the entire experiment with the analyzed time periods indicted and in (C), the corresponding time-averaged spectra for the control condition and drug treated state, respectively].

### Linking broadband shifts in LFP power spectra to underlying neuronal activity

As mentioned in the Introduction, it is not entirely clear how the underlying neuronal circuit activity is contributing to the metabolically coupled signals used in functional PET and MRI, but it is likely that both dendritic currents and spiking activity have an impact (Logothetis, 2008; Hillman, 2014). Indeed, in a handful of studies specifically investigating how spiking activity relates to changes in LFP characteristics, evidence has been presented pointing to power shifts in the broadband (non-oscillatory) part of the LFP power spectrum being strongly correlated to changes in neuronal spiking (see e.g., Manning et al., 2009; Guyon et al., 2021). Additionally, in other studies, authors have used the slope of the aperiodic LFP spectrum to assess changes in the excitatory-inhibitory balance in cortical networks (Lombardi et al., 2017; Chini et al., 2022). Based on these results, effectively linking spiking activity to LFP data, we have here characterized changes in the aperiodic LFP power spectrum as a proxy for underlying changes in neuronal activity (with respect to both power and slope by fitting curves of the type (*y* = 10*^A^*/f*^B^*) in the frequency interval 1–300 Hz to LFP power distributions where the two free parameters A and B were considered to represent changes in power and slope, respectively; Figures 1C, D).

### Characterizing broadband shifts in LFP power spectra

**FIGURE 2:**
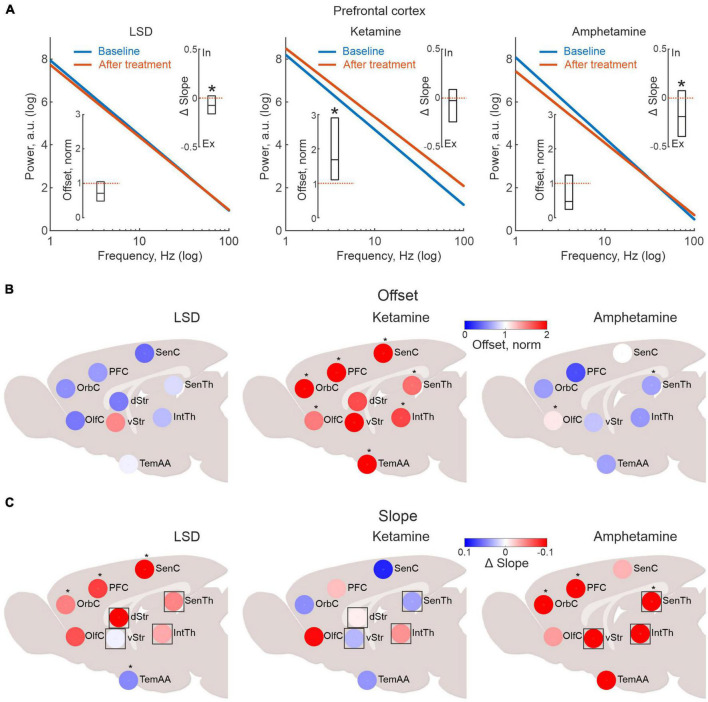
Lysergic acid diethylamide (LSD), ketamine and amphetamine treatment are associated with dissimilar brain activation patterns. **(A)** Linear fits in log-log scale illustrating the drug-induced changes in aperiodic local field potential (LFP) power for all electrode pairs located in the prefrontal cortex (blue line represents baseline and red after drug treatment). The inserted boxes denote the median offset and slope changes and their respective 25 and 75% percentiles (the corresponding values for all structures mapped are presented in panels 2 **(B,C)**. **(B)** Pharmacological imaging of LFP power changes indicating neuronal firing rate changes. In the presented maps, LFP data are congregated into nine larger structures to ensure sufficient coverage across animals. Color scale denotes median power offset from baseline (as indicated in Figure 1C). Note the clear differences in the mapped response patterns between ketamine, LSD and amphetamine. Scatter plots of the same data as in **(A)**, divided into within and between structure connectivity (black line indicate linear fit and red dotted line unity). **(C)** Pharmacological imaging of LFP slope changes indicating changes in excitatory-to-inhibitory (E-I) balance. Asterisks in panels **(A–C)** mark significant changes in the drug treated state compared to baseline values (*p* < 0.05). Regions marked with square symbols in **(C)**, lack internal populations of both excitatory and inhibitory neurons, suggesting external input may be contributing.

Investigating the effects of LSD, ketamine and amphetamine, we found that LFP power changes differed substantially between treatments (Figure 2A and see also Supplementary Figures 2, 3). In specific, for ketamine, the offset of the average LFP power spectrum (parameter A), was significantly increased compared to baseline suggesting increased neuronal activity in both sensorimotor and cognitive-limbic parts of the cortico-basal ganglia-thalamic system (only in striatum did the increase in LFP power not reach significance when compared to baseline values; *p* < 0.05; Figure 2B). This pattern was in stark contrast to the LSD-induced brain state, where activity levels, if anything, instead tended to be weakly decreased, but with none of the nine structures showing significant changes compared to baseline (*p* < 0.05). For comparison, the non-hallucinogenic psychostimulant amphetamine was also characterized, overall showing modest changes in power with significant changes observed only for olfactory cortex and sensory thalamus (up and down, respectively; *p* < 0.05).

Next, changes in slopes (parameter B in the exponential fits) were analyzed for the three treatments. However, when mapping the slope values across all recording sites, it should be kept in mind that not all brain structures contain distinct groups of excitatory and inhibitory neurons (specifically, the rodent thalamic nuclei lack inhibitory interneurons and striatum is void of excitatory interneurons). Non etheless, external input can still provide excitation or inhibition, even if both cell types are not present in the internal microcircuits. Thus, for completeness, all nine structures have been included in Figure 2C (for transparency, structures without both excitatory and inhibitory neurons in their internal circuitry are indicated by squares). These analyses revealed that LSD principally induced a flattening of the slope, indicating an E-I shift toward more excitation, as indicated by the warm colors in Figure 2C (with the exception of the temporal association area, asterisks denote significant changes compared to baseline; *p* < 0.05; Gao et al., 2017; Lombardi et al., 2017; Chini et al., 2022). Interestingly, amphetamine tended to change the slope in a similar way (with significant flattening in frontal cortex; *p* < 0.05), whereas no consistent pattern could be found after ketamine (and none of the regions displayed significant changes).

Thus, taken together our pharmacological mapping data suggest that the physiological brain states induced by LSD and ketamine, respectively, involve very different patterns of activation—at least for the features assessed by the current LFP-based metric.

### Characterizing shifts in functional connectivity

**FIGURE 3:**
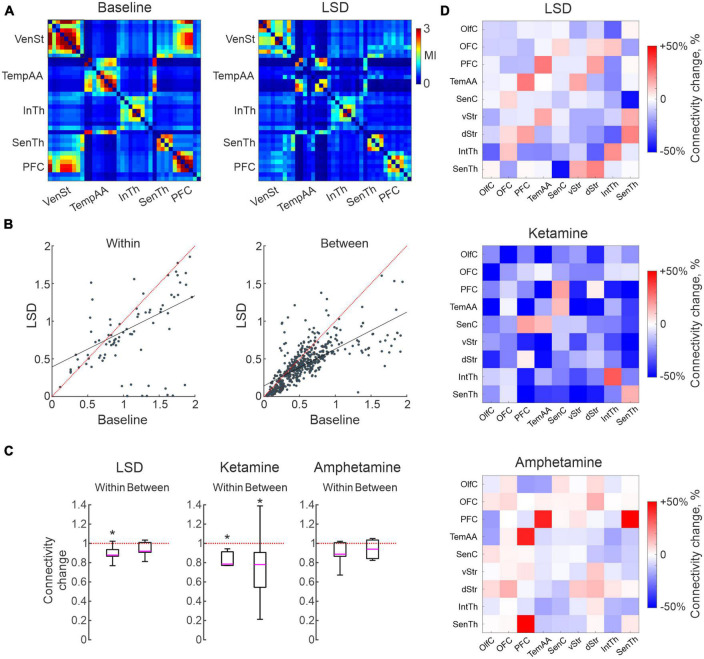
Characterizations of changes in functional connectivity based in measures of mutual information. **(A)** Connectivity matrix illustrating the connectivity strength for 38 electrodes located in five brain structures, from an example recording before/after lysergic acid diethylamide (LSD) treatment. Note a higher connectivity with in than between structures but with large variations, and a tendency for reduced connectivity following LSD treatment. **(B)** Scatter plots of the same data as in **(A)**, divided into within and between structure connectivity. **(C)** Boxplots illustrating global measures of reduction in connectivity. Asterisks mark significant changes (*p* < 0.05). **(D)** Connectivity matrices summarizing the average change in connectivity induced by the three treatments for each combination of the nine structures (cool colors represents reduction and warm an increase).

Human imaging studies have indicated changes in functional coupling between certain brain nodes as a key physiological characteristic of treatment with psychedelics (Scheidegger et al., 2012; Doss et al., 2022; Golden and Chadderton, 2022; Smausz et al., 2022) as well as ketamine (Alario and Niciu, 2021). Equivalent studies have, however, been difficult to carry out with high-resolution techniques in animal studies since it requires parallel recordings in extensively distributed neuronal networks. Making use of the multi-structure recording design developed for this study, we here applied time series analyses of monopolar LFP signals recorded from the 128 individual microelectrodes to address this question. Analyses of MI is a quite useful tool in this context. MI informs on the amount of information that can be obtained about one random variable by observing another random variable. MI is often used in neuroimaging analyses, including in fMRI studies, and is also used to assess brain connectivity (Wang et al., 2009; Ince et al., 2017; Yin et al., 2017; Sun et al., 2018). Indeed, functional connectivity based on MI has been demonstrated to reflect physiologically relevant features of the brain networks better than simple correlations under some conditions (Sun et al., 2018). Here, we applied the MI estimate proposed in Ince et al., 2017 for the LFP time series to characterize intra- and inter-structural connectivity changes caused by LSD, ketamine or amphetamine. We used all pairs of electrodes for which MI was calculated in 2 s windows during the 15 min baseline and after treatment episodes. It should perhaps be noted that estimates of functional connectivity using this measurement represents physiological processes on a much finer time scale (milliseconds rather than seconds) than similar metrics applied in PET/fMRI studies. Using this metric, we observed that connectivity differed substantially between pairs of electrodes recording already during baseline conditions. As expected, electrodes located in the same structure typically displayed higher connectivity to each other than pairs of electrodes in separate structures (mean difference 70%). Nevertheless, the range of connectivity values found for different pairs of electrodes within/between structures were, in fact, comparable indicating a higher degree of granularity than what the current, rather coarse, functional grouping may suggest. An overview of pairwise electrode connectivity values (baseline vs. LSD for 38 channels distributed in five structures) is shown for an example recording in Figure 3A. Overall, a decrease in functional connectivity was the dominating effect for all three treatments (of varying degree), and there was also a tendency for a roughly proportional decrease in relation to the electrodes initial connection strengths (although there were also several exceptions; R-squared for the linear fits of all recordings were for (within structure: 0.84 ± 0.08, 0.69 ± 0.21, 0.70 ± 0.15) and (between structures: 0.79 ± 0.09, 0.64 ± 0.30, 0.69 ± 0.18) for LSD, ketamine and amphetamine, respectively; Figure 3B). On a global level, the observed decreased functional connectivity reached significance for ketamine (both within and between structures) and for LSD (within structures; *p* < 0.05; Figure 3C).

Finally, to address the question if certain structures were more affected than others, we mapped the changes in connectivity from each of the nine subregions to all others in each of the recordings. Summarizing these analyses, the pattern of changes was not strikingly different for different pairs of regions (Figure 3D; although, of potential interest is perhaps the slight increases in connectivity observed for a few inter-structural connections, breaking the general pattern, involving PFC in all three treatments). Nevertheless, the general trend toward decoupling of functional connectivity at the structure-to-structure level will probably remain, although further studies will be needed to clarify more detailed connectivity changes on a finer spatial scale.

## Discussion

In this study we have characterized features of the LFP signal, relating to power, slope, and temporal correlations, in large-scale parallel recordings in rats treated with LSD, ketamine and amphetamine, under the supposition that these measures carry information concerning changes in neuronal activity, E-I balance and functional connectivity, respectively, (Manning et al., 2009; Ince et al., 2017; Lombardi et al., 2017). To date, the functional characterizations of hallucinogenic substances have largely been carried out using non-invasive brain imaging techniques such as fMRI. However, although it would be highly valuable to be able to relate the current finding to previous imaging data, direct comparisons are complicated by insufficient understanding of how various types of neuronal activity drives the hemodynamic response (Ekstrom, 2010). For example, pioneering work by Logothetis and co-workers suggested that the BOLD contrast mechanism primarily reflects the input and intracortical processing of a given area rather than its spiking output (Logothetis et al., 2001), however, later studies have also implicated neuronal spiking as a contributor to the BOLD signal, at least under certain experimental conditions (Kahn et al., 2013). In any event, because non-oscillatory LFP power is thought to be correlated to neuronal activity (Manning et al., 2009), it should also correlate with glucose and oxygen consumption—making this signal potentially useful as a physiological biomarker linking high-resolution electrophysiological recordings in animals to human imaging data—at least on longer time scales (seconds). A few technical limitations remain in the current experimental design that also deserves to be mentioned. First, although a large number of electrodes were implanted in each animal to achieve high-resolution recording on a close to global scale, all relevant brain structures can obviously not be covered in the same animal. Second, to get enough measurement points across animals and sufficient statistical power, electrodes were grouped together into nine larger functional groups. This compromises the initial very high spatial resolution (electrode spacing was 250 μm). Third, by repeating experiments many times in each rat matched data samples were obtained, which helps creating robust statistical comparisons but with the drawback that animals were not drug naïve. Although we found no evidence for carry-over effects between consecutive experiments, receptor desensitization could still have contributed at least to some extent to reducing the effect-sizes in our data. Moreover, translating findings between rodents and humans may have limitations for cognitive processes and conscious states, because the much larger human brain has the potential for much more complex interactions between large-scale networks (Bressler and Menon, 2010). At the same time, because several of the basic circuits recorded in the current study are known to be highly evolutionary conserved across mammalian species (Stephenson-Jones et al., 2011), it is reasonable to assume that the main findings of the current study relating to drug induced physiological changes in cortico-basal ganglia-thalamic circuits should be translatable across different mammalian species, including humans.

The present findings lend support to certain previously published results in animal studies, but do not fully adhere to previous articles. For example, while ketamine induced a robust power increase, indicative of increased neuronal activity and higher firing rates (Jackson et al., 2004), this change was not associated with a shift in E-I balance, as judged by the LFP slope. This is not in accordance with the notion that the blockage of NMDA receptors on fast-spiking interneurons leads to a disinhibition of pyramidal cells causing the general increase in firing rates (Suzuki et al., 2002; Homayoun and Moghaddam, 2007; Wood et al., 2012; Furth et al., 2017; Amat-Foraster et al., 2019; for a review on this topic see e.g., Rotaru et al., 2012). It is, however, of potential importance in this context that we here selectively analyzed the non-oscillatory part of the LFP spectrum, which clearly favors asynchronous spiking over, for example, gamma-entrained spiking activity (Guyon et al., 2021). On the other hand, the LFP slope and power analyses suggested that LSD (as well as amphetamine) causes a shift in E-I balance toward excitation, without an associated change in neuronal activity. This somewhat unexpected combination of findings is, however, well in line with certain published results. For example, while serotonin 2A agonists have been shown to increase glutamatergic transmission in prefrontal cortex, these observations have not been associated with direct evidence of concomittant increases in firing rates (Aghajanian and Marek, 1997, 1999, 2000; Zhou and Hablitz, 1999; Béïque et al., 2007). Future studies with recordings of well isolated single units will, however, be needed to verify if the observed LFP changes do indeed represent the proposed changes in rate and E-I balance.

Finally, in the present study, an almost global tendency for reduced functional connectivity, was observed for both ketamine and LSD. This result may appear surprising, given that animal experiments have demonstrated synchronous and functionally coupled high-frequency oscillatory activity in several frontal structures after treatment with both NMDA antagonists (Hunt et al., 2006, 2011; Hakami et al., 2009; Nicolás et al., 2011; Kulikova et al., 2012) and serotonin 2A agonists (Goda et al., 2013; Hansen et al., 2019). Notably, however, while the high-frequency oscillatory activity clearly is a conspicuous phenomenon following LSD and ketamine treatment, these oscillations nevertheless make up a rather limited part of the full LFP spectrum with relatively lower amplitude compared to low-frequency activity. This finding may also be related to the increase in entropy reported by some groups (Schartner et al., 2015, 2017; Timmermann et al., 2019).

In the current study, connectivity analysis was performed in the time-domain, meaning that while certain oscillatory components were also included, the main contribution to the signals was nevertheless aperiodic components of the LFPs (indeed, in control experiments, low-pass filtering of the data to eliminate the conspicuous HFOs observed >100 Hz was carried out, which resulted in virtually identical connectivity measures).

Overall, in this perspective, we believe the current study, which is primarily centered on aperiodic features of the LFP, can complement other studies in clarifying the physiological effects of psychedelics on the brain (Brys et al., 2022).

With these data at hand, the field will be able to move faster toward safer and more efficient therapeutic use of these compounds without risking potential backlashes caused by unforeseen side effects.

## Data availability statement

The raw data supporting the conclusions of this article will be made available by the authors, without undue reservation.

## Ethics statement

This animal study was reviewed and approved by Malmö/Lund Ethical Committee of animal experiments.

## Author contributions

SB and IB designed and performed experiments and reviewed the manuscript. PH designed experiments, analyzed the data, and reviewed the manuscript. AN analyzed the data, produced the figures and statistics, and reviewed the manuscript. PP designed experiments, analyzed the data, and wrote the manuscript draft. All authors contributed to the article and approved the submitted version.

## Footnotes

1
ClinicalTrials.gov

## Supplementary material

Supplementary Figure 1 Photo of the microelectrode array, consisting of 128 individual tungsten wires (33 μm in diameter and insulated with formvar) used for the LFP recordings.

Supplementary Figure 2 Examples of averaged spectrograms representing the differential LFP signal from pairs of electrodes located in PFC in conjunction with LSD **(left panel)** and amphetamine **(right panel)** treatment. **(Bottom panels)** show the corresponding time averaged spectra for the 30 min time periods indicated with white bars in spectrograms. White vertical dashed line marks time of injection; black and magenta lines for the two spectra represent fits of the form (y = 10^A^/f^B^) to the non-oscillatory part of the data.

Supplementary Figure 3 Individual examples of linear fits in log-log scale illustrating the drug-induced changes in aperiodic LFP power for electrode pairs located in the sensorimotor cortex, sensory thalamus and ventral striatum [blue line represents baseline and red after the drug treatment (LSD, ketamine or amphetamine)].
