# Supplementary figures and images for: Systems-level analysis of local field potentials reveals differential effects of lysergic acid diethylamide and ketamine on neuronal activity and functional connectivity
Source: Front Neurosci. 2023 May 23;17:1175575. doi: 10.3389/fnins.2023.1175575 (PMC10242129; doi:10.3389/fnins.2023.1175575)

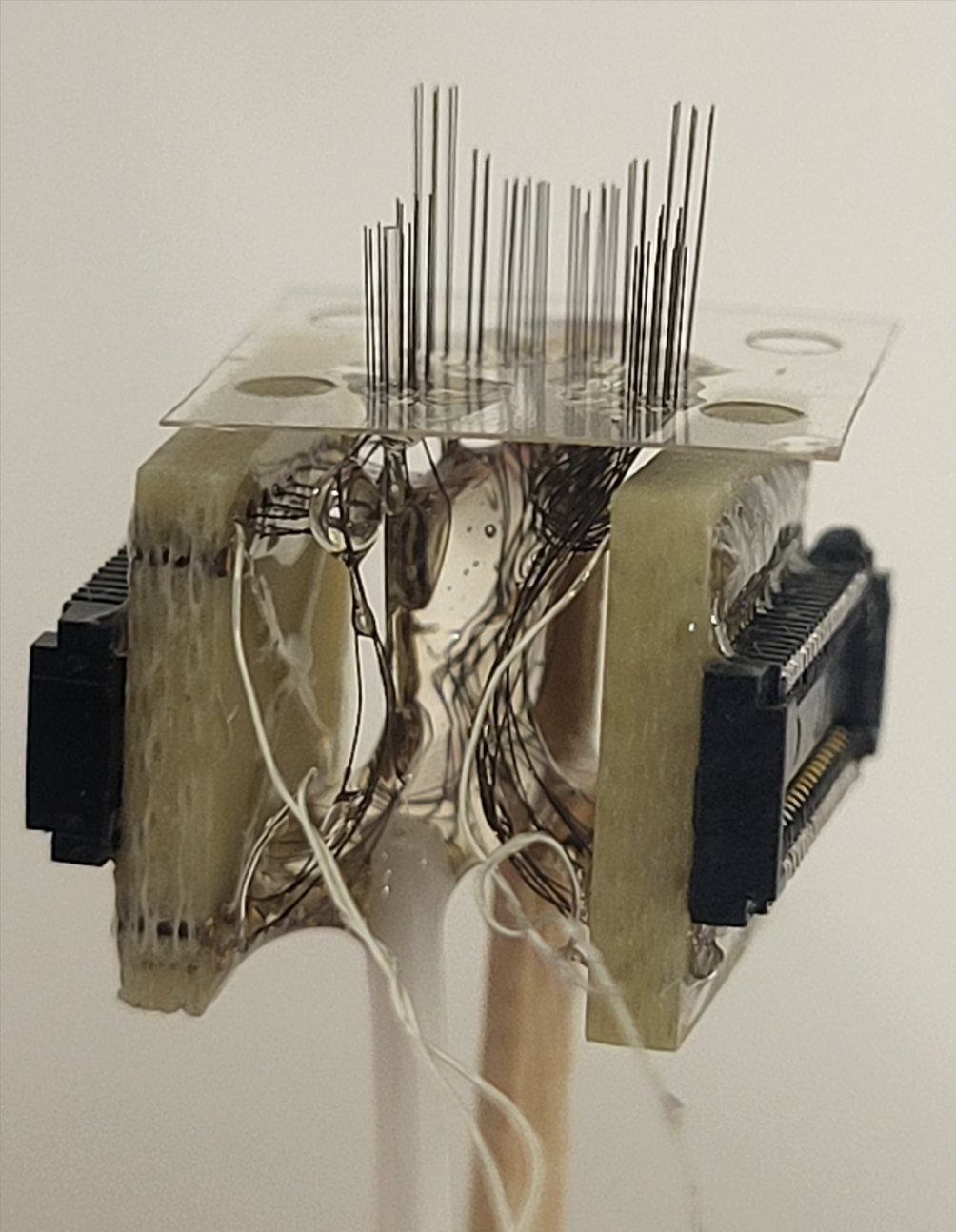

Supplement: Supplementary Figure 1 — Photo of the microelectrode array, consisting of 128 individual tungsten wires (33 μm in diameter and insulated with formvar) used for the LFP recordings. [file Image_1.TIF]

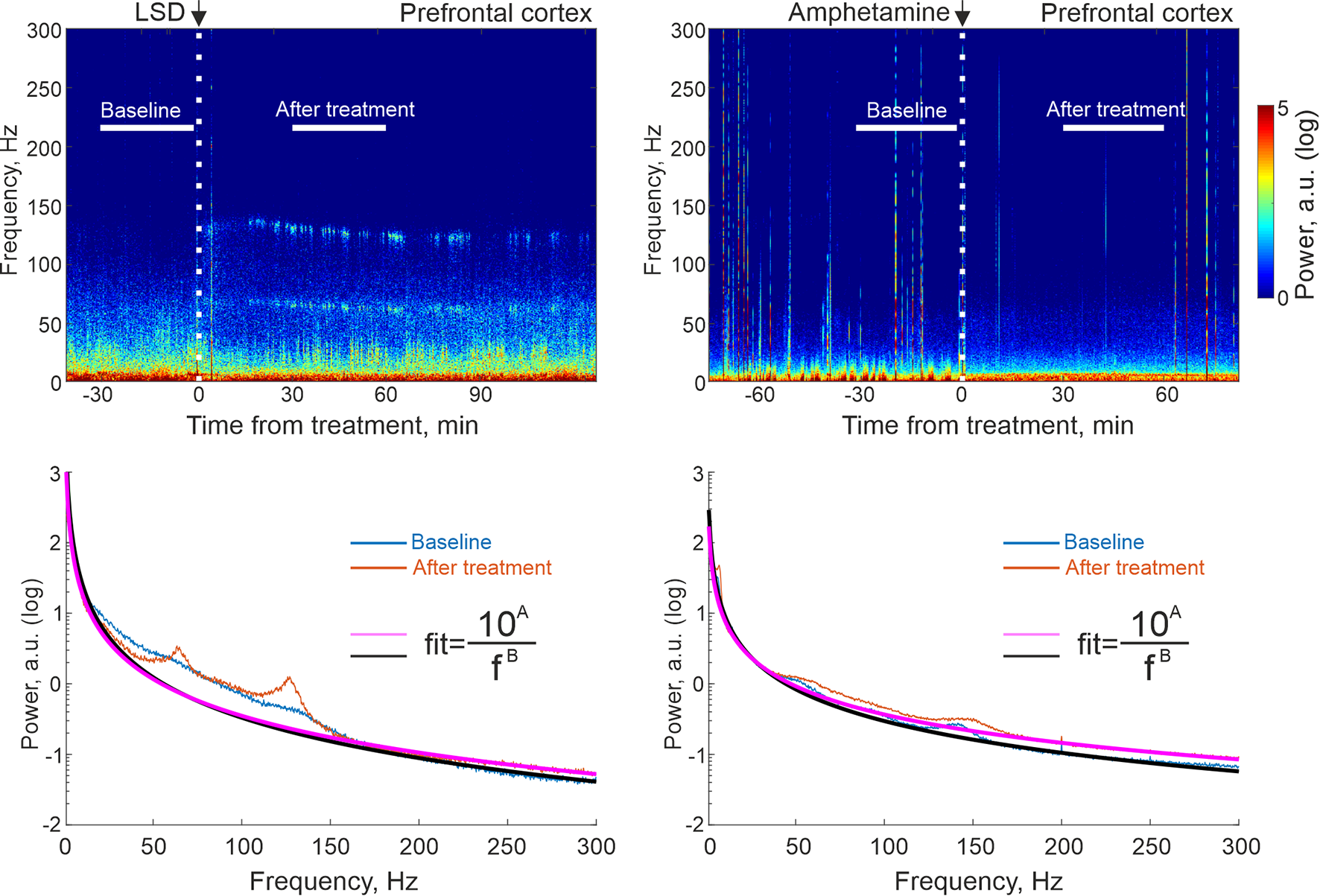

Supplement: Supplementary Figure 2 — Examples of averaged spectrograms representing the differential LFP signal from pairs of electrodes located in PFC in conjunction with LSD (left panel) and amphetamine (right panel) treatment. (Bottom panels) show the corresponding time averaged spectra for the 30 min time periods indicated with white bars in spectrograms. White vertical dashed line marks time of injection; black and magenta lines for the two spectra represent fits of the form (y = 10A/fB) to the non-oscillatory part of the data. [file Image_2.TIF]

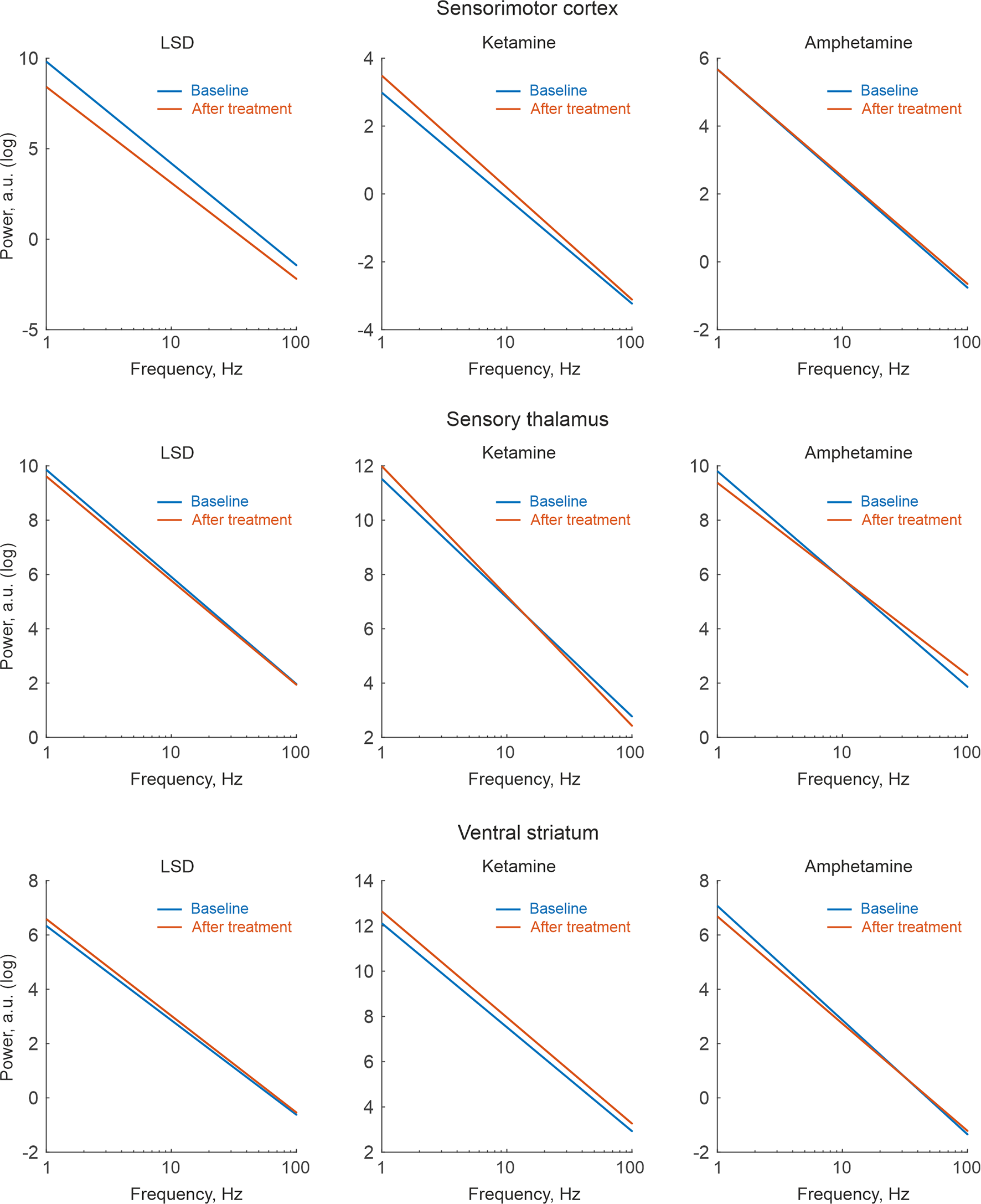

Supplement: Supplementary Figure 3 — Individual examples of linear fits in log-log scale illustrating the drug-induced changes in aperiodic LFP power for electrode pairs located in the sensorimotor cortex, sensory thalamus and ventral striatum [blue line represents baseline and red after the drug treatment (LSD, ketamine or amphetamine)]. [file Image_3.TIF]
